# Biostimulant Capacity of *Chlorella* and *Chlamydopodium* Species Produced Using Wastewater and Centrate

**DOI:** 10.3390/biology11071086

**Published:** 2022-07-20

**Authors:** Ainoa Morillas-España, Ángela Ruiz-Nieto, Tomás Lafarga, Gabriel Acién, Zouhayr Arbib, Cynthia V. González-López

**Affiliations:** 1 Department of Chemical Engineering, University of Almería, 04120 Almería, Spain; ame778@ual.es (A.M.-E.); angelaruiznieto@gmail.com (Á.R.-N.); lpt365@ual.es (T.L.); facien@ual.es (G.A.); 2 Functional Desalination and Photosynthesis Unit, CIESOL Solar Research Centre, 04120 Almería, Spain; 3 Sustainability Area FCC Aqualia, 04001 Almería, Spain; zouhayr.arbib@fcc.es; 4 Research Center for Mediterranean Intensive Agrosystems and Agrifood Biotechnology CIAIMBITAL, 04120 Almería, Spain

**Keywords:** microalgae, biostimulants, gibberellins, auxins, wastewater, biomass

## Abstract

**Simple Summary:**

The world population is expected to grow by over 2 billion people in the coming decades, involving an increase in agricultural production. Agriculture demands huge amounts of water and energy, so it is crucial to minimise the use of these resources to ensure a sustainable future. Plant biostimulants can promote germination, plant growth, flowering, and crop productivity, as well as increase nutrient-use efficiencies and resistance to abiotic stress. Microalgae are a novel and interesting source of biostimulants, and they can grow using wastewater. Although there is great interest in developing and applying these natural biostimulants produced from microalgae, there is still only a limited number of well-characterised and stable products available commercially. It is therefore necessary to identify novel strains that have a biostimulant capacity that are robust, that can grow in wastewater, and that are highly productive. This work determines the viability of producing high-quality microalgal biomass using wastewater and assesses the biostimulant capacity of the produced biomass. It is focused on an initial laboratory-scale study to produce these strains in wastewater and a preliminary validation of their biostimulant capacity.

**Abstract:**

The aim of the present study was to assess the potential of producing four microalgal strains using secondary-treated urban wastewater supplemented with centrate, and to evaluate the biostimulant effects of several microalgal extracts obtained using water and sonication. Four strains were studied: *Chlorella vulgaris* UAL-1, *Chlorella* sp. UAL-2, *Chlorella vulgaris* UAL-3, and *Chlamydopodium fusiforme* UAL-4. The highest biomass productivity was found for *C. fusiforme*, with a value of 0.38 ± 0.01 g·L^−1^·day^−1^. *C. vulgaris* UAL-1 achieved a biomass productivity of 0.31 ± 0.03 g·L^−1^·day^−1^ (the highest for the *Chlorella* genus), while the N-NH^4+^, N-NO_3_^−^, and P-PO_4_^3−^ removal capacities of this strain were 51.9 ± 2.4, 0.8 ± 0.1, and 5.7 ± 0.3 mg·L^−1^·day^−1^, respectively. *C. vulgaris* UAL-1 showed the greatest potential for use as a biostimulant—when used at a concentration of 0.1 g·L^−1^, it increased the germination index of watercress seeds by 3.5%. At concentrations of 0.5 and 2.0 g·L^−1^, the biomass from this microalga promoted adventitious root formation in soybean seeds by 220% and 493%, respectively. The cucumber expansion test suggested a cytokinin-like effect from *C. vulgaris* UAL-1; it was also the only strain that promoted the formation of chlorophylls in wheat leaves. Overall, the results of the present study suggest the potential of producing *C. vulgaris* UAL-1 using centrate and wastewater as well as the potential utilisation of its biomass to develop high-value biostimulants.

## 1. Introduction

The world population is expected to grow by over two billion people in the coming decades. Being able to feed this increasing population without further endangering the environment or depleting the world’s natural resources and biodiversity is a great scientific and technological challenge [1]. In the past, the most common strategy to increase agricultural production was to expand land usage. However, today, agriculture occupies approximately 38% of the Earth’s terrestrial surface [2] and is responsible for the disappearance of approximately half of the world’s forests [3]. For this reason, current practices now aim to increase productivities rather than land usage. Agriculture also demands huge amounts of water and energy, and so it is crucial to minimise the use of these resources to ensure a sustainable future. Accordingly, there are high hopes for the implementation of a circular economy [4].

Plant biostimulants can promote germination, plant growth, flowering, and crop productivity, as well as increase nutrient-use efficiencies and resistance to abiotic stress [5]. They are one of the top trends in agriculture and are being widely used in organic cropping systems. Microalgae are a novel and interesting source of biostimulants. Different microalgal strains from the genera *Chlorella*, *Acutodesmus*, *Arthrospira*, *Scenedesmus*, *Dunaliella*, and *Anabaena* have demonstrated their biostimulant capacity in scientific studies. For example, increased nutrient absorption and improvements in abiotic stress tolerance have been observed as well as a clear improvement in crop yield and quality [6]. The biostimulant activity of microalgae and microalgal extracts is associated with their primary metabolites (carbohydrates, proteins, and lipids), amino acids such as proline, arginine, and tryptophan, as well as vitamins, glycine betaine, and polysaccharides such as β-glucans [7]. Their compositions include various hormones such as auxins, gibberellins, and cytokinins, which, despite being present in small quantities, provide the algal extracts with marked effects that translate into various agronomic benefits [8]. Microalgae production and microalgae-derived products offer many advantages, such as reduced environmental impact and widespread consumer acceptance [9,10]. Moreover, these microorganisms can be produced on nonarable land and using different types of water and nutrient sources, including agricultural waste and urban wastewater. The main challenge is to reduce the high production cost of the microalgal biomass. To achieve this, it has been suggested that using wastewater and flue gases as the nutrient sources is a key strategy [11]. Using wastewater was initially proposed as an alternative for reducing production costs. However, it is now also considered as an alternative to using conventional wastewater treatment processes, which are polluting and require large amounts of energy. Microalgae have the capacity to recover (not just remove) the nutrients present in wastewater while simultaneously minimising greenhouse gas emissions, saving energy, and producing valuable bioproducts [12,13]. The microalgae produced can be used as a feedstock for valuable agricultural products such as biopesticides and the above-mentioned biostimulants, or to produce aquafeeds that have a proven effectiveness in increasing food sustainability and animal welfare [14].

Microalgal biomass can potentially prevent nutrient loss by gradually releasing nitrogen, phosphorus, or potassium into the environment. Various microalgae-based biofertilisers and biostimulants are currently commercially available; these include AlgaFert^®^ and AlgaFert Eco^®^, marketed by the Spanish company Biorizon Biotech SL (Almería, Spain). One of the main challenges that arises in producing metabolites of interest for agriculture is the lack of adequate characterisation of these biostimulants, which is essential for them to perform reliably in the market. There is also a lack of knowledge regarding the biostimulatory mechanisms of these bioproducts, making it difficult to design tailored organic farming strategies that predict their effects on crops, especially when applied under different temperature and humidity conditions. To facilitate the diffusion of these products throughout the agricultural sector, knowledge of the exact composition of the bioproducts should be improved along with the standardisation of the production processes and the elucidation of the molecular and physiological mechanisms of action [15]. Microalgae have also been suggested as biological biocides to manage pests and diseases [16]. Ongoing studies and developments in this field will certainly result in commercial microalgae-derived biocides appearing in the future.

Although there is great interest in developing and applying these natural biostimulants produced from microalgae, there is still only a limited number of well-characterised and stable products available commercially. It is therefore necessary to identify novel strains that have a biostimulant capacity, that are robust, that can grow in wastewater, and that are highly productive. Consequently, the objective of this research is to determine the viability of producing high-quality microalgal biomass using wastewater and to assess the biostimulant capacity of the produced biomass. The use of UAL microalgal strains as biostimulants has not yet been evaluated. Therefore, this work focuses on an initial laboratory-scale study to produce these strains in wastewater and a preliminary validation of their biostimulant capacity.

## 2. Materials and Methods

### 2.1. Selected Microorganisms

The microalgal strains studied are available at the culture collection of the Department of Chemical Engineering of the University of Almeria in Spain. The selected strains were UAL-1 (*Chlorella vulgaris*), UAL-2 (*Chlorella* sp.), UAL-3 (*Chlorella vulgaris*), and UAL-4 (*Chlamydopodium fusiforme*), all of which have proven effectiveness as biofertilisers (Ranglová et al., 2021). The inocula were produced using 1 L controlled photobioreactors at 25 ± 1 °C, 100 µmol photons·m^−2^·s^−1^, and maintained using a modified Arnon medium, as described elsewhere [17].

### 2.2. Biomass Production

The culture medium was prepared using secondary-treated wastewater supplemented with centrate. The secondary treatment of the urban wastewater was performed using a pilot-scale thin-layer cascade photobioreactor containing a microalgae–bacteria consortium dominated by *Scenedesmus* sp. [18]; this water was ultrafiltered for later use using a 10 m^2^ Bio-Cell BC10 ultrafiltration membrane (Ecotec S.A., Barcelona, Spain). Because of the high efficiency of this initial step, the wastewater used had a low nutrient content: N-NH_4_^+^ 1–5 mg·L^−1^; N-NO_3_^−^1–5 mg·L^−1^, and P-PO_4_^3−^ 1–5 mg·L^−1^. For this reason, centrate was added until a total nitrogen concentration of 180 mg·L^−1^ was reached. The centrate used as the main nutrient source was kindly provided by El Bobar wastewater treatment plant (Almería, Spain).

Biomass production was performed using controlled 0.30 L bubble columns with spherical bases filled with 0.25 L of culture [19]. Each reactor was aerated at 0.2 v/v/min and the pH was controlled and kept constant at 8.0 by the on-demand injection of pure CO_2_. Illumination was provided by eight 28 W Daylight T5 fluorescent tubes (Phillips, Madrid, Spain). The tubes were located horizontally 1 cm apart from each other and 4 cm away from the cultures. The illumination was carried out using a 12:12 h light/dark cycle that simulated outdoor conditions, namely, a progressive increase in light intensity from 08:00 am to 02:00 pm and a progressive decrease from 02:00 pm to 08:00 pm. The average irradiance in the centre of the columns, which were filled with the culture medium alone, was 780 μmon photons·m^−2^ s^−1^. A spherical quantum sensor (SQS-100, Walz GmbH, Effeltrich, Germany) was used to determine the light intensity. The temperature of the cultures was maintained at 25 °C by controlling the temperature of the whole room.

The reactors were inoculated with 10% of the culture volume from a standard inoculum at a concentration of 1.0 g·L^−1^. The reactors were initially operated in batch mode until the stationary phase was reached, after which they were operated in semicontinuous mode using a dilution rate of 0.3 day^−1^ for 10–11 days. This was the period required to replace the total volume of the reactors at least twice and achieve a constant biomass concentration for a minimum of three consecutive days. The harvested microalgal biomass and the supernatant obtained after centrifuge separation were frozen at −20 °C until further analysis.

### 2.3. Assessment of the Biomass Productivity and Nutrient Consumption

The biomass concentration was measured on a dry-weight basis after drying the biomass in an oven at 80 °C for 24 h. The biomass productivity (grams of biomass produced per litre of culture per day) was calculated as the product of the biomass concentration and the dilution rate (0.3 day^−1^). The F_v_/F_m_ ratio, which is an indicator of the maximum quantum yield of the PSII chemistry was measured daily using an AquaPen AP 100 fluorimeter (Photon System Instruments, Drásov, The Czech Republic). The microalgal cells were dark-adapted for 5 min prior to taking the measurements.

The standard official methods approved by the Spanish Ministry of Agriculture were used to assess the nitrogen and phosphorus composition of the culture media and supernatant once the biomass was harvested. Briefly, N-NO_3_^−^ was quantified using a spectrophotometer to measure absorbance at 220 and 275 nm, N-NH_4_^+^ was measured using the Nessler reactive method, and P-PO_4_^3+^ was measured through the phospho–vanado–molybdate complex using visible spectrophotometry. The nutrient content of the media (after removing the biomass) was analysed at the beginning (day 0), prior to inoculation, at the end of the batch phase, and at the end of the semicontinuous production. The determinations were conducted in triplicate.

### 2.4. Biomass Processing

To obtain the biostimulant extracts, a cell wall disruption step was required. In the present study, cell disruption was carried out by sonicating the biomass using a UP 400 S ultrasonic processor (Hielscher Ultrasonics, Teltow, Germany). Briefly, 0.2 L of microalgal sludge were sonicated in continuous mode at 24 kHz with a FC22K flow cell (Hielscher Ultrasonics, Teltow, Germany). The ultrasonic processor has a variable power output control; this was set at 70% during the experiments, which lasted for 5 min. A magnetic stirrer was used to guarantee sample homogenization. The dry weight of the disrupted biomass was obtained by drying it in an oven at 100 °C for 48 h; this value was then used to calculate the dilutions needed to obtain microalgal extract concentrations of 0.1, 0.5, and 2.0 g·L^−1^.

### 2.5. Assessment of the Biostimulant Capacity

#### 2.5.1. Germination Index Assessment of Watercress Seeds (*Lepidium sativum* L.)

The gibberellin-like effect of the extracts was determined by assessing the germination index (*GI*) of the watercress seeds. The method used has been described elsewhere [8]. Microalgal extracts with biomass concentrations of 0.5 and 0.1 g·L^−1^ were prepared and compared against a control (distilled water). To assess the *GI*, 25 commercial watercress seeds were placed on Whatman No. 5 filter paper and put inside a sterilised Petri dish. Bioassays were conducted in triplicate using three sterilised Petri dishes per treatment (75 seeds per treatment). The Petri dishes were treated with 2 mL of distilled water or microalgal extracts. The seeds were allowed to grow for 3 days at 24 °C in the dark and the *GI* was then determined by *N* (the number of germinated seeds) multiplied by *L* (the average length of the germinated seeds) divided by *N_c_* (the number of germinated seeds treated only with distilled water) and multiplied by *L_c_* (the average length of the germinated seeds when treated only with distilled water). The total was multiplied by a hundred to obtain the percentage.

$$GI\left(\%\right)=\frac{N\cdot L}{N_{c}\cdot L_{c}}\cdot 100$$

#### 2.5.2. Assessment of the Adventitious Soybean (*Glycine max* L.) Root Induction

The auxin-like effect of the extracts was assessed by determining adventitious root induction using commercial soybeans. Briefly, the soybeans were planted at a depth of 1 cm in moistened perlite. These were maintained in a growth chamber at 27 °C controlled under a 12/12 h light/dark cycle. After 7 days of incubation, two seedlings were cut (3 cm below the cotyledon) and placed in vials containing 20 mL of the corresponding microalgal extract. The cut seedlings were incubated at 27 °C (12 h light/dark) for 7 days. After this period, the adventitious roots on each hypocotyl that were longer than 1 mm were counted. This number increases proportionally to the auxin concentration of the sample. Four vials for each extract were placed in the chamber, so eight seedlings were counted per extract concentration. Indol-3-butyric acid (IBA, a specific auxin) was used as a positive control. The results were expressed as the percentage of variation with respect to the samples treated only with distilled water (the negative control).

#### 2.5.3. Excised Cucumber (*Cucumis sativus* L.) Expansion Test

The cytokinin-like effect of the extracts was determined using commercial cucumber seeds. These were placed on glass trays with 0.7% agar-solidified Knop nutrient medium containing 450 mL of distilled water, 3.5 g of bacteriological agar, and 50 mL of KNOP solution (5 g·L^−1^ Ca (NO_3_)_2_·4H_2_O, 1.25 g·L^−1^ KNO_3_, 1.25 g·L^−1^ KH_2_PO_4_, and 1.25 g·L^−1^ MgSO_4_·7H_2_O). Then, the trays containing the seeds were transferred to an incubator and kept at 27 °C in darkness for 5 days. After this period, 5 uniform cotyledons were weighed using a precision balance and transferred to 60 mm Petri dishes containing a wet filter paper with 3 mL of one of the following solutions: distilled water (the negative control), 6-benzylaminopurine (BAP, the positive control), or microalgal extract (two different concentrations were studied: 0.5 and 2.0 g·L^−1^). Four Petri dishes were used for each extract concentration, so 20 cotyledons were weighed in total. The results are expressed as the percentage of weight variation with respect to the samples treated with distilled water [20].

#### 2.5.4. Wheat (*Triticum aestivum* L.) Chlorophyll Retention Assay

The commercial wheat seeds were first rinsed in tap water for 4 h. They were then planted at a 1 cm depth in moistened perlite. The trays were placed in a growth chamber (25 °C, 65% relative humidity) that was illuminated with fluorescent lamps, where they remained for 10 days (12 h light/darkness). The leaves from the seedlings (approximately 10 cm long) were excised in 10 mm segments, cut 3 cm from their apical tip. The fresh weight of ten segments was measured with an analytical balance and placed in 50 mL vials containing 10 mL of either distilled water (the negative control), 6-benzylaminopurine (BAP, the positive control), or the microalgal extracts (at concentrations of 0.5 or 2.0 g·L^−1^). Four vials were used for each extract concentration so that 40 segments were weighed in total. The vials were placed back inside the controlled chamber, where they remained for 4 days. Subsequently, the leaves were blot dried and put into graduated 15 mL tubes containing 8 mL of 80% ethanol in distilled water (*v*/*v*). The test tubes were transferred to a water bath at 80 °C, and, after 10 min, the solution was chilled using an ice bath. The extracts were centrifuged, and the optical density determined at 645 nm using a spectrophotometer. The optical density was normalised to 100 mg fresh weight and the adjusted results were compared to the control [21].

### 2.6. Statistical Analysis

The microalgae were cultivated in three independent experimental units (photobioreactors) with three technical replicates being taken per natural replicate. The pH, temperature, and illumination were controlled and monitored online. In all cases, the fixed factor was the culture media. The normality and the homoscedasticity of the variables within each group were checked. The results were analysed by ANOVA using Statgraphics v.18 software (Statgraphics Technologies Inc., The Plains, VA, USA). Duncan’s multiple range test was used to identify differences between samples.

## 3. Results and Discussion

### 3.1. Biomass Productivity

**Figure 1:**
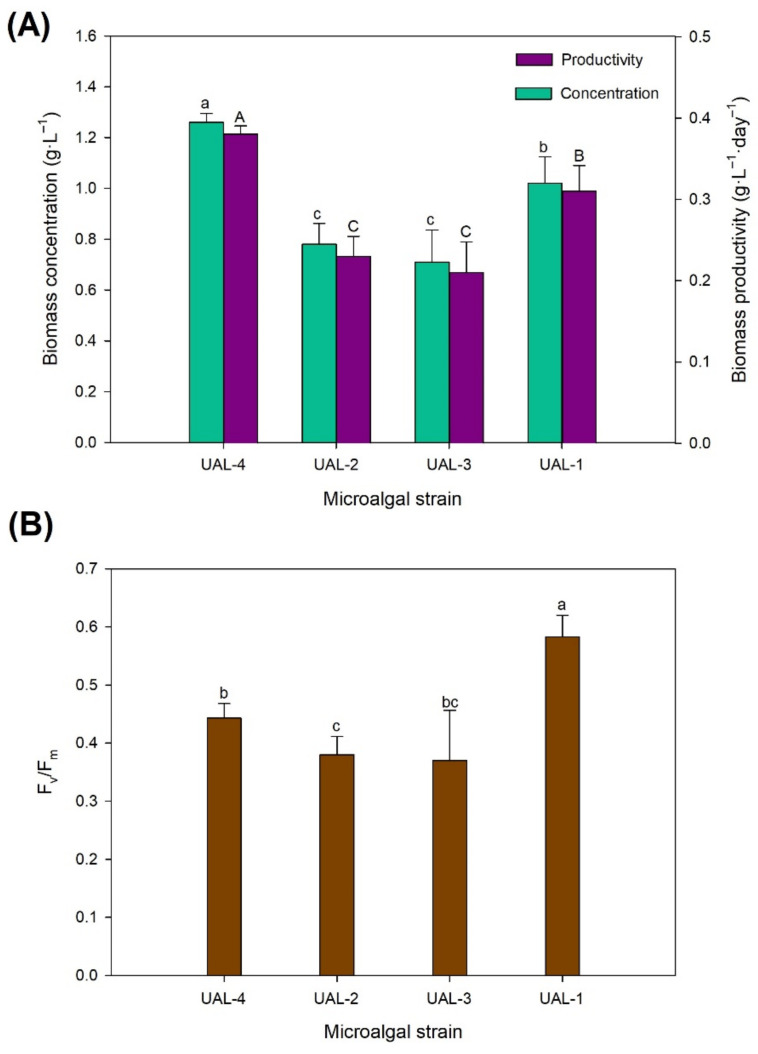
Effect of the culture media on (**A**) biomass productivity and (**B**) maximum quantum yield of the PSII chemistry. Values represent the mean values ± SD. Different letters indicate significant differences (*p* < 0.05).

Only a limited number of microalgal strains achieve commercial success. Their success can be attributed to different factors, but mainly it is down to their capacity to produce valuable products and that these products can be produced using large outdoor reactors [22]. Another factor affecting a strain’s commercial success is its production costs; these are determined by the culture medium used and the downstream processing required [23]. A further attribute needed for a microalgal strain to achieve commercial success is that it is fast-growing and highly productive. When cultivated in wastewater, it is important to select a strain that grows rapidly, thus enabling greater nutrient removal and increased wastewater treatment process efficiency. The biomass productivities of the selected strains are shown in Figure 1. Overall, the choice of the inoculated strain significantly affected the biomass concentration and the biomass productivity (*p* < 0.05). The highest concentration (1.26 ± 0.03 g·L^−1^) and the highest biomass productivity (0.38 ± 0.01 g·L^−1^·day^−1^) were achieved when producing the microalga UAL-4 (*p* < 0.05). Similar values were reported by Ruiz et al. [24] for *Chlorella vulgaris* when grown in wastewater, with the final biomass concentration ranging from 712 to 1300 mg SS·L^−1^; differences in the final biomass concentration were related to differences in the nutrient loads. The strains UAL-1, UAL-2, and UAL-3 achieved biomass concentrations of 1.02 ± 0.10, 0.78 ± 0.08, and 0.71 ± 0.12 g·L^−1^, respectively, and productivity values of 0.31 ± 0.03, 0.23 ± 0.02, and 0.21 ± 0.03 g·L^−1^·day^−1^, respectively. These results are in line with those reported for other strains when produced using wastewater or waste streams as the nutrient sources [25].

Previous works conducted on the microalga UAL-4 revealed that, when produced using freshwater and pure nutrients, the biomass concentration could reach 1.9 ± 0.1 g·L^−1^ [26]. The lower values reported here could be attributable to operating the reactors in semicontinuous mode—in the above-mentioned study, the experiments were carried out in batch mode. The culture medium composition might also have contributed to these results. A commonly assumed general composition for microalgae is the Redfield ratio (16), which suggests that N:P molar ratios of 16:1 in the culture media are optimal for microalgal growth [27]. In the present study, the N:P ratio in the inlet effluents was 11–12, suggesting the culture might be phosphorus-limited. The Redfield ratio is not a universal biochemical optimum; rather, it represents a suitable starting point average and can predict potential nitrogen or phosphorus limitation [28]. In our study, besides the inorganic nutrients, the wastewater and centrate could have contained other compounds that might have limited microalgal growth. Indeed, the F_v_/F_m_ values (Figure 1) were lower than the theoretical optimum value (0.7) for eukaryotic microalgae [29]. The F_v_/F_m_ values ranged from 0.37 to 0.58 and were lower in the UAL-2 and UAL-3 cultures; these were also the strains with the lowest biomass productivity. The low values indicate that the cultures were subjected to some type of stress, which might have been caused by the presence of a compound inhibiting algal growth. The nitrogen content in the centrate is usually high and so the leachate needs to be diluted because nitrogen is normally present as N-NH_4_^+^, which can indeed inhibit algal growth [30].

The microalga UAL-1 was better able to adapt to the wastewater and centrate and thus showed greater potential for being produced in large outdoor photobioreactors. The use of ultrafiltration membranes has proven to be a good strategy for treating wastewater with microalgae, as it allows one to treat a greater volume and avoids centrifugation, which is more expensive. Therefore, the process will be scaled up in future works to evaluate the use of membrane systems, their process costs, and their sustainability.

### 3.2. Nutrient Consumption

**Figure 2:**
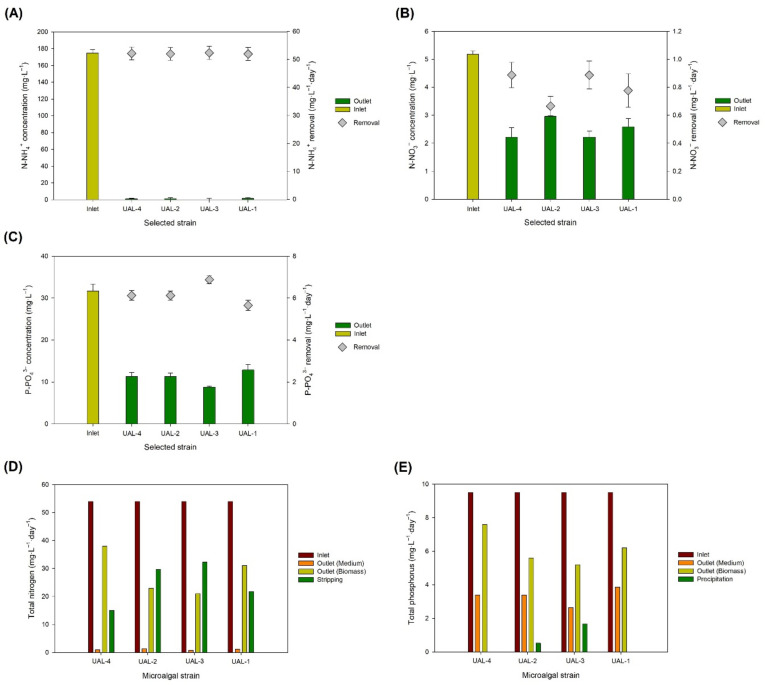
Concentration and daily removal of (**A**) N-NH_4_^+^, (**B**) N-NO_3_^−^, and (**C**) P-PO_4_^3−^, along with the mass balance of (**D**) nitrogen and (**E**) phosphorus. Values represent the mean values ± SD.

When producing microalgae using waste streams and wastewater, the main goal, apart from the microalgae production itself, is to recover nutrients and to allow the safe disposal of the treated water into the environment. Nitrogen and phosphorus derived from natural activities (fertilisers, wastewaters, animal wastes, etc.) are a major concern because they are harmful to health and to the environment (causing eutrophication). Most of the nitrogen contained in wastewater is in the form of organic nitrogen and N-NH_4_^+^; consequently, nitrites and nitrates are produced during wastewater treatment. A secondary aim of the current study was to assess the potential of the selected strains to recover nitrogen and phosphorus from the supplemented wastewater. The results obtained for the four strains are shown in Figure 2. In all cases, the N-NH_4_^+^ was completely removed from the media, with removal values higher than 50 mg L^−1^ day^−1^ (Figure 2A). This does not mean that the N-NH_4_^+^ was assimilated by the microalgae—some of it could have been transformed into N-NO_3_^−^ by the action of nitrifying bacteria, and some could have been lost to the atmosphere by desorption. The N-NO_3_^−^ concentration in the outlet effluents was also lower than in the inlets (possibly due to consumption by microalgae), with N-NO_3_^−^ removal rates of 0.89, 0.67, 0.89, and 0.78 mg·L^−1^·day^−1^ for UAL-4, UAL-2, UAL-3, and UAL-1, respectively. A mass balance of the reactor, assuming a 10% nitrogen content in the biomass, revealed that not all the N-NH_4_^+^ was used by the microalgae and bacteria to produce biomass (Figure 2D). Ammonia stripping or desorption was responsible for around 25–55% of the total nitrogen removal. It is important to highlight that the mass balance was conducted assuming an identical nitrogen content in the biomass; moreover, the organic nitrogen compounds in the wastewater were not considered. Therefore, the results are approximate, but they do demonstrate that stripping occurs during wastewater treatment using microalgae even if the medium’s temperature and pH are controlled. Overall, the outlet nitrogen concentration was in the 2–3 mg·L^−1^ range, below the maximum discharge limit (10–15 mg·L^−1^) set by Spanish and EU regulations [31].

Regarding phosphorus (P-PO_4_^3−^), the removal rates observed are shown in Figure 2C. The highest P-PO_4_^3−^ removal was achieved using UAL-3 at a rate of 6.8 mg·L^−1^·day^−1^, followed by a comparable removal rate of nearly 6 mg L^−1^day^−1^ for UAL-4, UAL-2, and UAL-1. Because of the high initial P-PO_4_^3−^ inlet concentration, the outlets still retained a relatively high P-PO_4_^3−^ concentration and did not comply with existing regulations, which set the maximum discharge limit at 1–2 mg·L^−1^ [31]. For this reason, it would be necessary either to decrease the dilution rate in order to increase the hydraulic retention time, and thus allow greater nutrient consumption, or to supplement the medium with N-NO_3_^−^ so as to increase microalgal growth and therefore increase the P-PO_4_^3−^ uptake by the microalgal cells. It is important to note that the present study aimed to select one strain for large-scale production rather than to optimise the process. Process optimisation must be conducted independently for each photobioreactor design and location. This is possible because laboratory-scale photobioreactors are now suitable for wastewater treatment at the commercial scale. Nevertheless, incorrect pH control must be avoided, as it can cause phosphorus precipitation and reduced phosphorus availability, leading to lower biomass productivity. In this work, the pH in the reactors was monitored online and the phosphorus mass balance revealed that phosphorus precipitation was minimal. The mass balance was carried out assuming a phosphorus content in the biomass of 1.5%. Different microalgae have different compositions and, therefore, the results shown in Figure 2E are only an estimation. However, they demonstrate that the pH was correctly controlled, since low phosphorus precipitation (or even no precipitation) was observed, depending on the strain.

Previous works have suggested that microalgae-based wastewater treatment processes could recover up to 90% of the total nutrients while reducing the energy consumption by half compared to conventional treatments [12]. Our results are in line with those studies, given that a large amount of the nutrients present in the culture medium were either assimilated by the microalgae or stripped into the atmosphere. However, the results also suggest that the N/P ratio of the medium is of key importance when it comes to complying with maximum discharge limits. When the phosphorus concentration is high, the wastewater’s nitrogen content will need to be adjusted using commercial nutrients to ensure safe disposal or effluent reutilisation.

### 3.3. Biostimulant Activity

**Figure 3:**
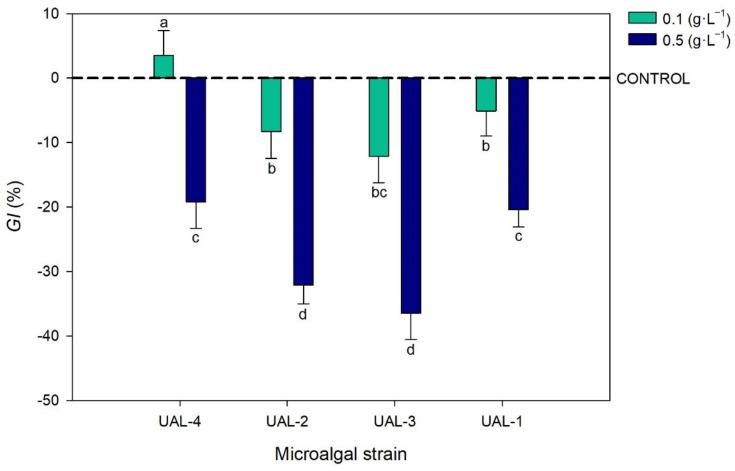
Effect of the microalgal extracts on the germination index of watercress seeds. Values represent the percentage of variation with respect to the control (distilled water). Values represent the mean values ± SD. Different letters indicate significant differences (*p* < 0.05).

One of the most interesting uses for microalgal biomass is the production of agricultural products, some of which are already available on the market. Microalgae-derived biostimulants encourage plant growth and development in a variety of ways; hence, it is necessary to assess the biostimulant effects using different bioactivity assays. Figure 3 shows the effects of the microalgal extracts on the germination index (GI) of watercress seeds at concentrations of 0.1 and 0.5 mg·L^−1^. The results are expressed as the percentage of GI variation with respect to the control (distilled water, 0%). All the microalgal strains and extract concentrations studied had a negative effect on the GI of the seeds, except for UAL-4 at a concentration of 0.1 g·L^−1^. The results reported here suggest the presence of bioactive compounds exhibiting gibberellin-like activity. Gibberellins are a group of plant hormones that play an important role in initiating seed germination and stem elongation [32]. The GI of the seeds was influenced both by the microalgal strain (*p* < 0.05) and the extract concentration (*p* < 0.01). Higher biomass concentrations led to reduced GI values, with an average decrease of 20% for UAL-4 and UAL-1, and 35% for UAL-2 and UAL-3. These results are consistent with previous reports indicating a negative correlation between watercress seed germination and the concentration of *Scenedesmus* sp. extracts [8,9,10,11,12,13,14,15,16,17,18,19,20,21,22,23,24,25,26,27,28,29,30,31,32,33]. In addition, a previous work revealed that bioactive molecules, such as abscisic acid (ABA), could produce shoot growth suppression (by themselves) and form an inhibitory complex along with other biomolecules, such as lunularic acids [34]. Moreover, the biostimulant compounds are produced inside the microalgae cells, so a cell disruption step is required to liberate the bioactive compounds [35]. In the above-mentioned study, the authors disrupted microalgal cells using high-pressure homogenisation. They reported that a slight improvement in the GI of the seeds could be achieved using milder disruption conditions [8]. Through microscopic observations, we could determine that the sonication step used in the current study completely disrupted the cells. Therefore, in future studies, milder disruption conditions will be imposed on the produced biomass.

**Figure 4:**
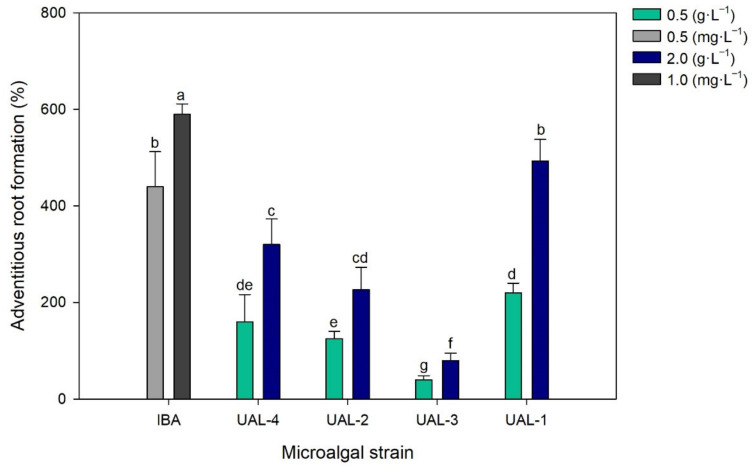
Effect of the microalgal extracts on the formation of adventitious roots. Values represent the percentage of variation with respect to the control (distilled water). Values represent the mean values ± SD. Different letters indicate significant differences (*p* < 0.05).

Auxins play a significant role in inducing root initiation and elongation. Figure 4 shows the development of mung bean roots. The results are expressed as a percentage comparison between the negative control (distilled water) and the positive control (auxin indole-3-acetic acid, IBA). All the extracts promoted growth, although the best result was obtained with UAL-1 at a concentration of 2.0 g·L^−1^; this led to a root development of 493%, which was not significantly different to that of IBA at 0.5 mg·L^−1^ (590%). Overall, the results suggest that the produced extracts exhibited potential biostimulant effects; for example, the UAL-4 strain at a concentration of 2.0 g·L^−1^ resulted in an increase of 320% compared to distilled water. These results are consistent with previous studies that demonstrated the biostimulant effects of a mixed microalgal consortium containing *Chlorella* sp., *Scenedesmus* sp., *Spirulina* sp., and *Synechocystis* sp. on tomato [36]. This could be because of more bioactive compounds in the microalgal biomass with hormone-like activity, which promotes root formation (or more precisely, the initiation of root formation) and elongation during the plants’ early developmental stages.

**Figure 5:**
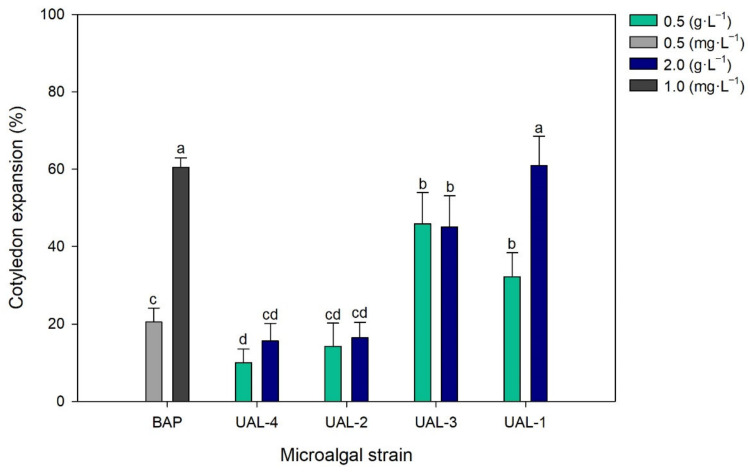
Effect of the microalgal extracts on the weight of cucumber cotyledons. Values represent the percentage of variation with respect to the control (distilled water). Values represent the mean values ± SD. Different letters indicate significant differences (*p* < 0.05).

Cytokinins are another group of phytohormones that control not only cell division but also bud and leaf-blade development. The cytokinin-like activity of the extracts was assessed using the cucumber cotyledon expansion test. Figure 5 shows the cotyledon expansion when using microalgal extracts compared to the negative control (distilled water, 0%). The values obtained are higher than the control for all four strains. In the case of UAL-1 and UAL-3, the values were even higher than those obtained with the BAP hormone at 0.5 mg·L^−1^, being 32.3% and 45.9% higher than the negative control. The results suggest that the microalgal extracts have a biostimulant effect. For example, at a concentration of 2.0 g·L^−1^, the UAL-1 strain led to an increase of 60.9% compared to distilled water. The values were higher than those reported for other natural extracts, such as those obtained by Navarro-López et al. [33] using a commercial biofertiliser made from algae or from *Scenedesmus obliquus* biomass without any pretreatment. This might be because of more bioactive compounds having hormone-like activity, perhaps as a result of the strain produced or the culture medium, either of which might affect the biomass composition.

**Figure 6:**
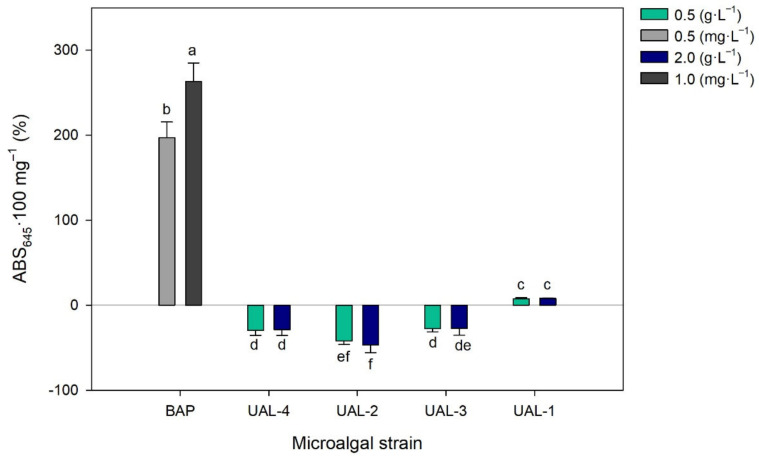
Effect of the microalgal extracts on the chlorophyll content of the detached wheat leaves. Values represent the percentage of variation in the ABS_645_/100 mg ratio with respect to the control (distilled water). Values represent the mean values ± SD. Different letters indicate significant differences (*p* < 0.05).

Finally, the chlorophyll retention test was carried out using wheat seeds to determine the cytokinin-like activity of the microalgal extracts. Figure 6 shows that only the UAL-1 strain presented positive values with respect to the control, being 7.5 and 8.0% higher at concentrations of 0.5 and 2 g·L^−1^, respectively. The results suggest that the UAL-1 strain has the highest content of biomolecules with biostimulant activity. However, when compared to the positive control, the results obtained for the microalgal extracts were significantly lower, even for UAL-1, despite being assessed at a much lower concentration. The use of wastewater as the nutrient source to produce microalgae and biostimulants is an interesting option that needs to be further validated at the large scale. Future works will optimise not only the biomass production but also the cell wall disruption step needed to obtain the biostimulant compounds contained inside the microalgal cells, and the conservation of the extract.

## 4. Conclusions

The results show that it is possible to produce the selected strains (*Chlorella vulgaris* UAL-1, *Chlorella* sp. UAL-2, *Chlorella vulgaris* UAL-3, and *Chlamydopodium fusiforme* UAL-4) using secondary wastewater supplemented with centrate. The *Chlorella vulgaris* UAL-1 strain obtained the highest biomass concentration compared to the other strains studied and is therefore a potential candidate for large-scale production. The results also revealed that this strain is a potential source of biostimulant compounds, thus increasing its potential industrial implementation in wastewater treatment processes. Furthermore, the UAL-1 microalga improved the germination index of watercress seeds, promoted the formation of adventitious soybean roots, and increased the chlorophyll content of wheat leaves, along with other benefits to the plants. It is important to highlight that the microalgae concentration of the evaluated extracts greatly influenced the biostimulant effect; hence, the application of these extracts must be optimised to ensure their efficacy. To confirm the results reported here, the biomass production process needs to be scaled up and the biostimulant effects verified in field trials.

## Data Availability

Not applicable.
